# Supplementary material for: Impact of Oseltamivir Treatment on Influenza A and B Virus Dynamics in Human Volunteers
Source: Front Microbiol. 2021 Mar 1;12:631211. doi: 10.3389/fmicb.2021.631211 (PMC7957053; doi:10.3389/fmicb.2021.631211)
Supplement: Supplementary file 3 [file Data_Sheet_3.PDF]

Impact of oseltamivir treatment on influenza A and B virus dynamics in human volunteers

Kyla L. Hooker and Vitaly V. Ganusov

Supplemental information

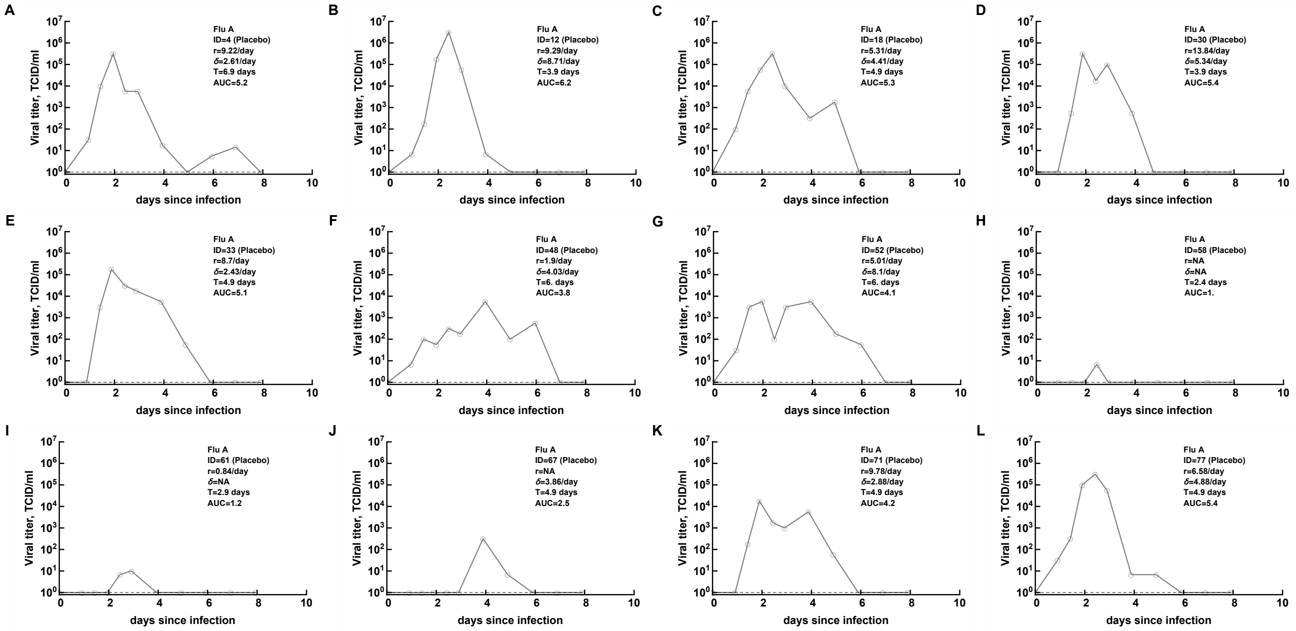

Figure S1: Viral shedding titers for individual volunteers from Flu A study [12]. These are data for placebo-treated volunteers including the volunteer ID, duration of infection, viral growth and viral decline rates, duration of infection, and the total viral sheeting (AUC). Volunteers excluded from the analysis as uninfected have the following IDs: 7, 21, 38, 41.

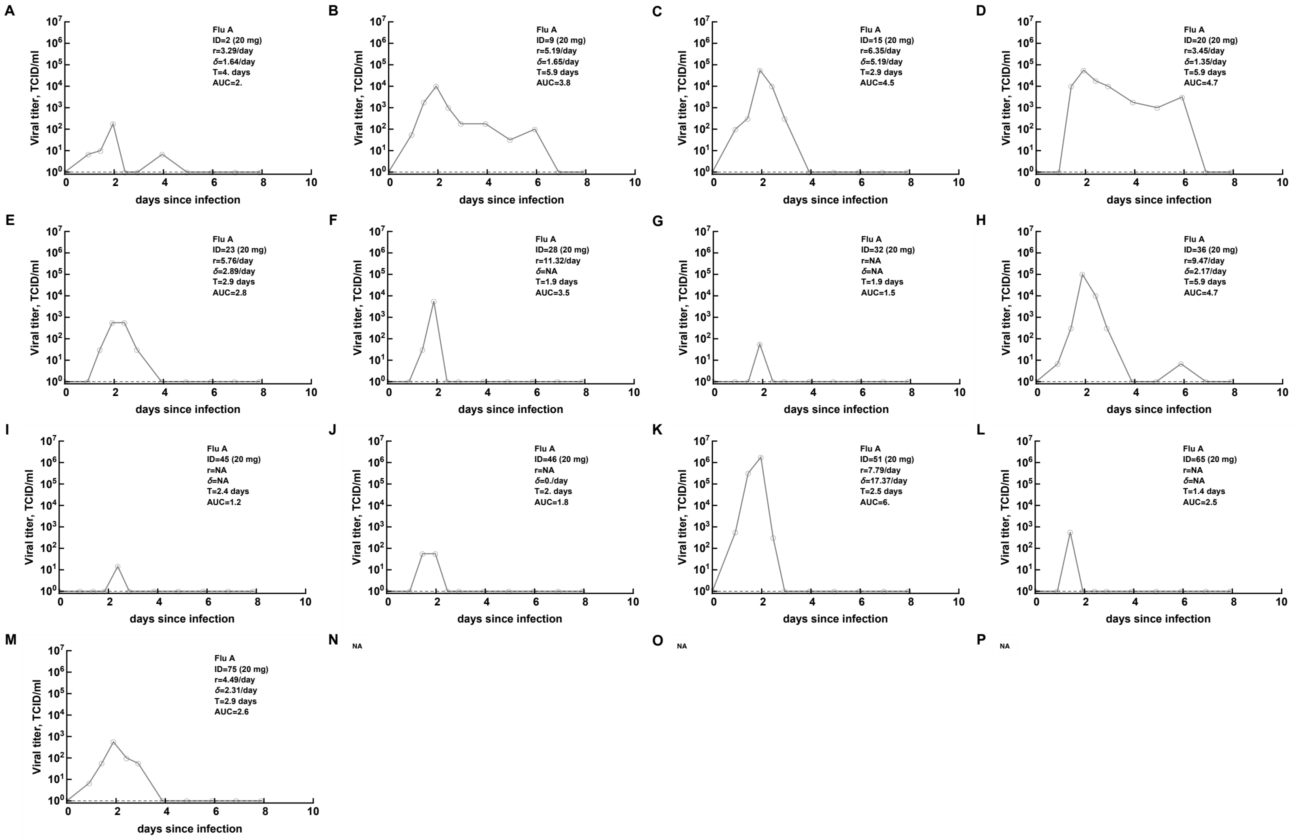

Figure S2: Viral shedding titers for individual volunteers from Flu A study [12] treated with 20mg of oseltamivir. Volunteers excluded from the analysis as uninfected have the following IDs: 56, 68, 79. See Figure S1 for more detail.

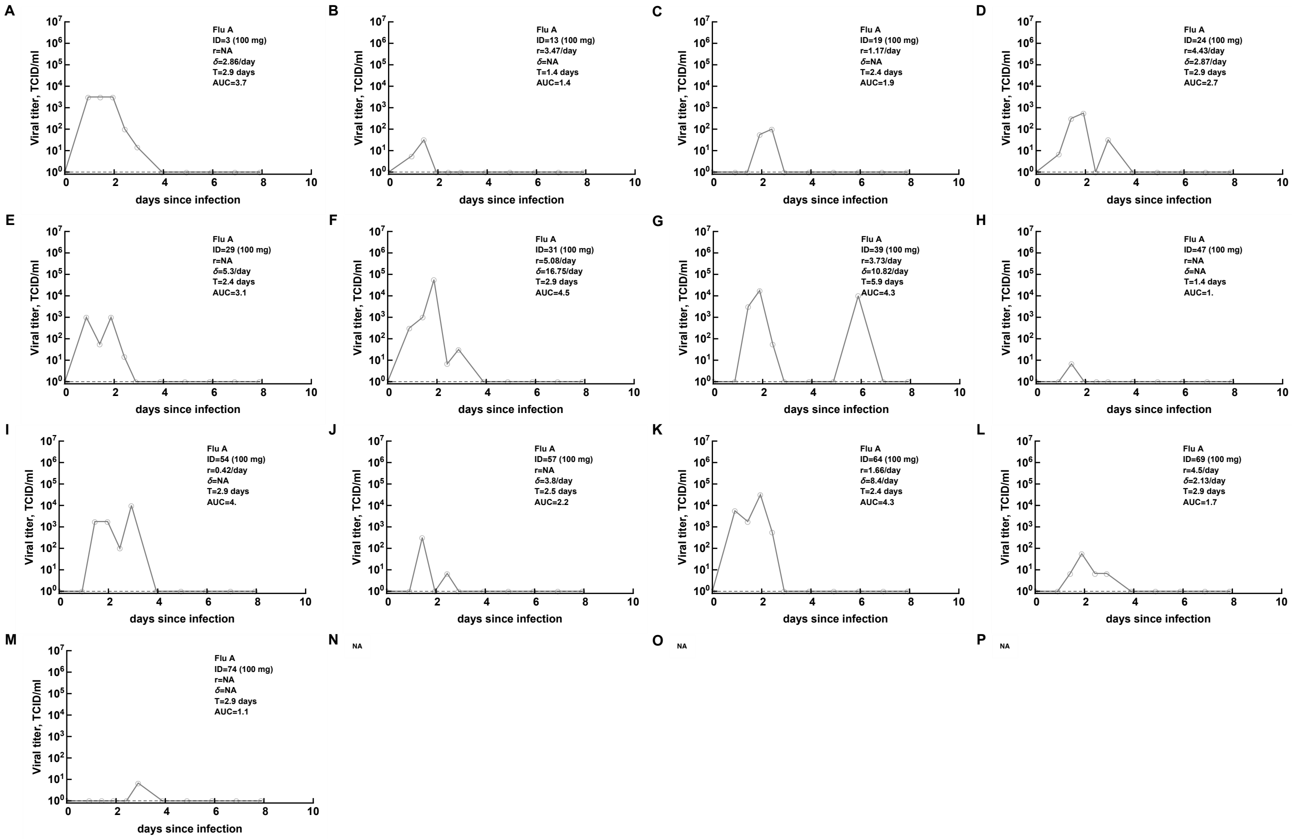

Figure S3: Viral shedding titers for individual volunteers from Flu A study [12] treated with 100mg of oseltamivir. Volunteers excluded from the analysis as uninfected have the following IDs: 10, 42, 78. See Figure S1 for more detail.

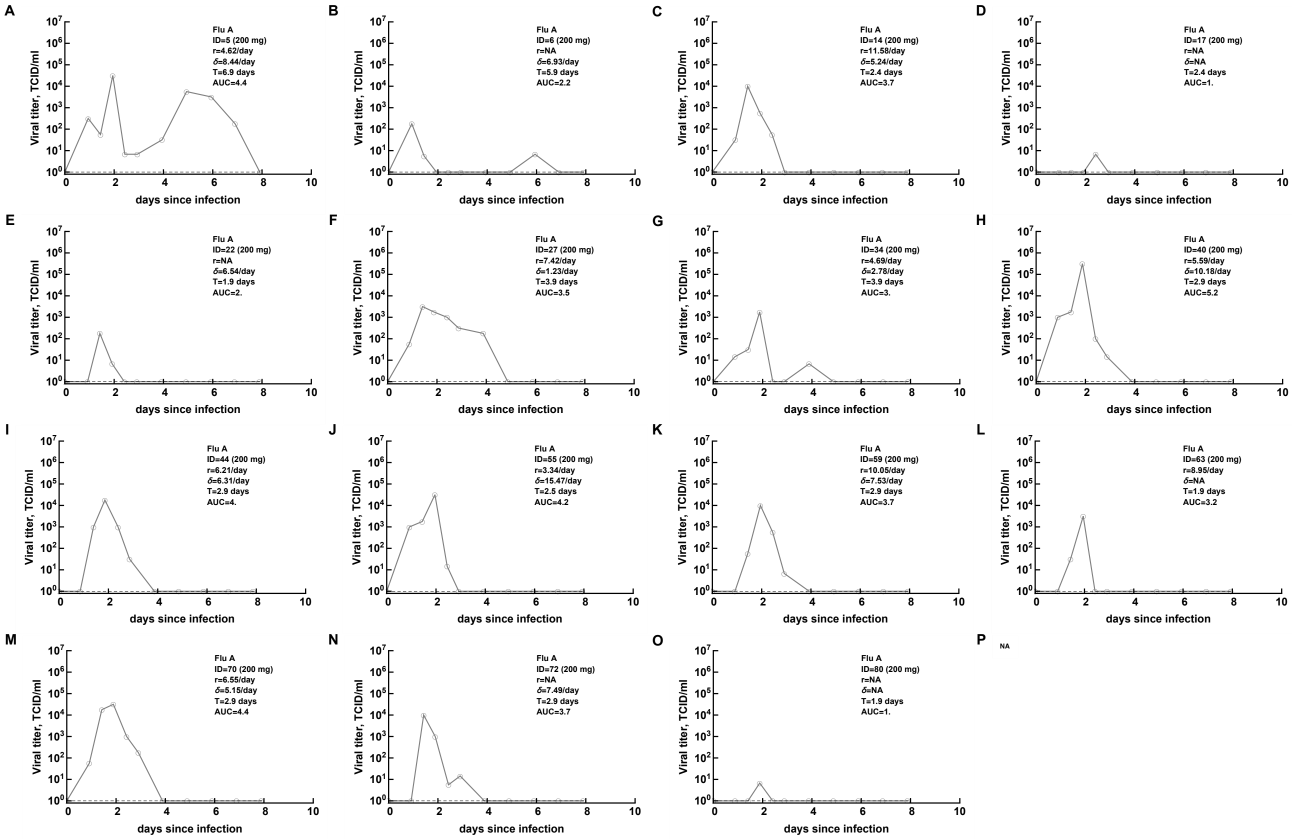

Figure S4: Viral shedding titers for individual volunteers from Flu A study [12] treated with 200mg of oseltamivir twice daily. Volunteers excluded from the analysis as uninfected have the following IDs: 50. See Figure S1 for more detail.

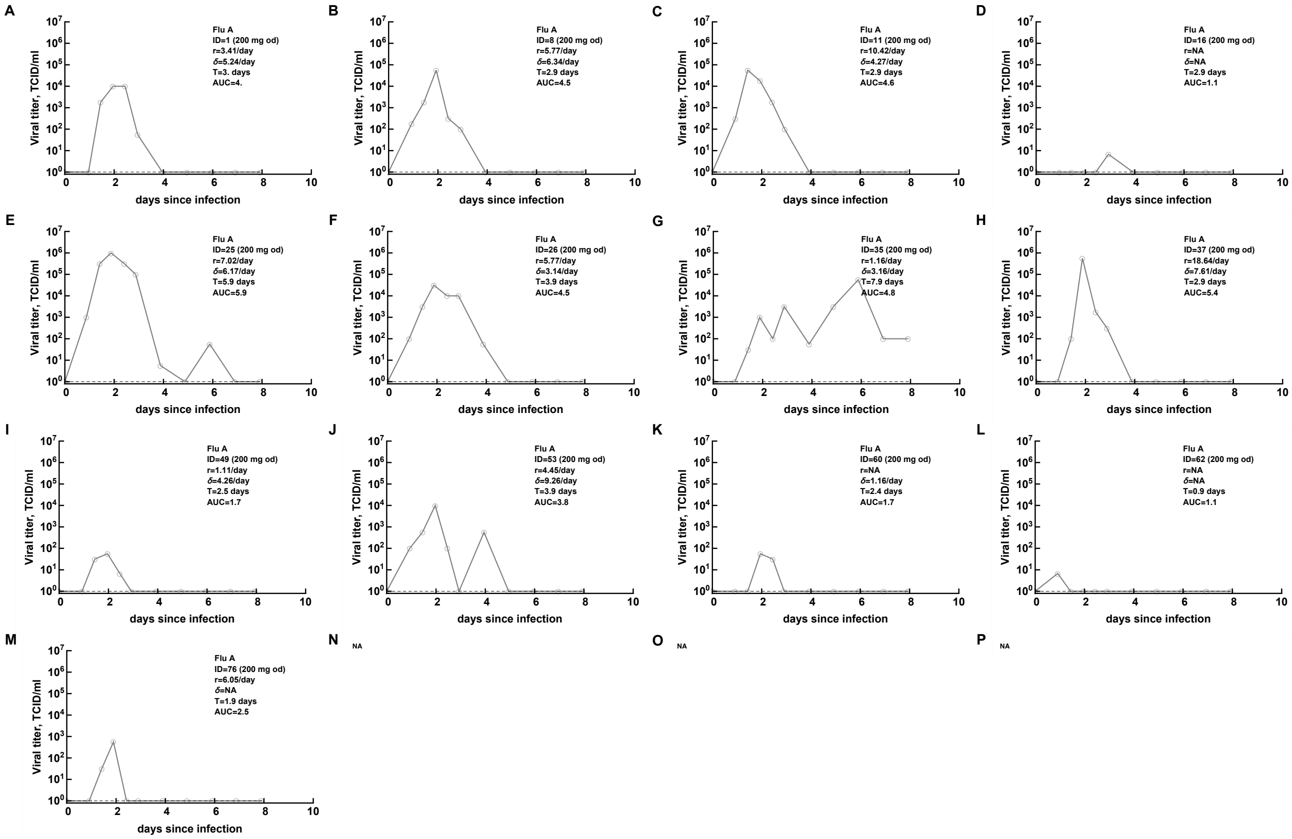

Figure S5: Viral shedding titers for individual volunteers from Flu A study [12] treated with 200mg of oseltamivir once daily. Volunteers excluded from the analysis as uninfected have the following IDs: 43, 66, 73. See Figure S1 for more detail.

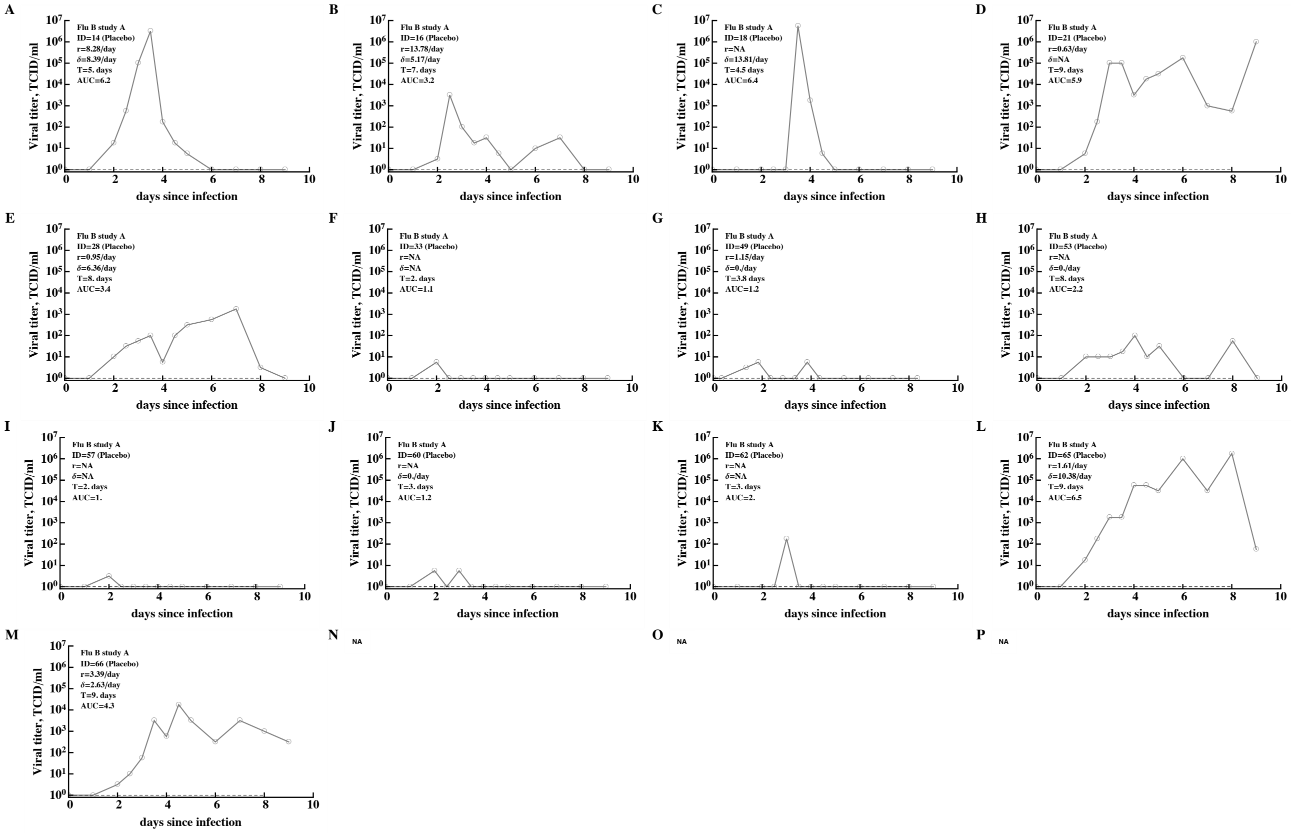

Figure S6: Viral shedding titers for individual volunteers from Flu B study A [13]. These are data for placebo-treated volunteers including the volunteer ID, duration of infection, viral growth and viral decline rates, duration of infection, and the total viral sheeting (AUC). Volunteers excluded from the analysis as uninfected have the following IDs: 25, 31, 35, 36, 41, 44, 47.

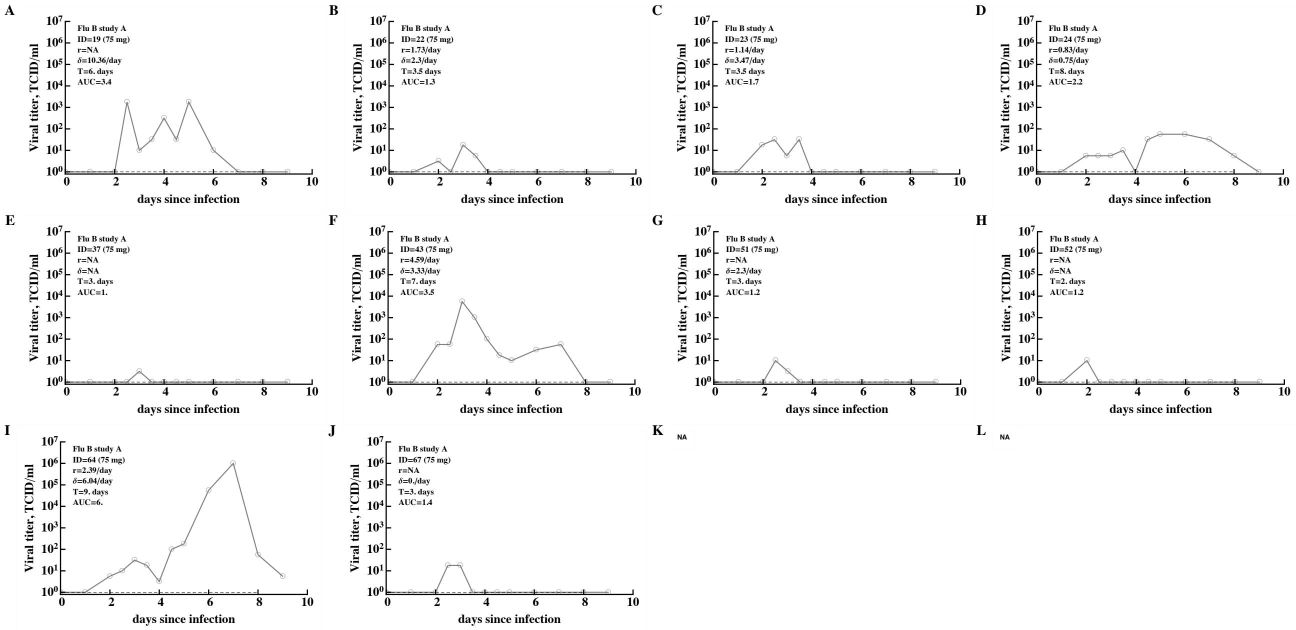

Figure S7: Viral shedding titers for individual volunteers from Flu B study A [13] treated with 75mg of oseltamivir. Volunteers excluded from the analysis as uninfected have the following IDs: 12, 13, 30, 32, 39, 45, 55, 58, 59, 70. See Figure S6 for more detail.

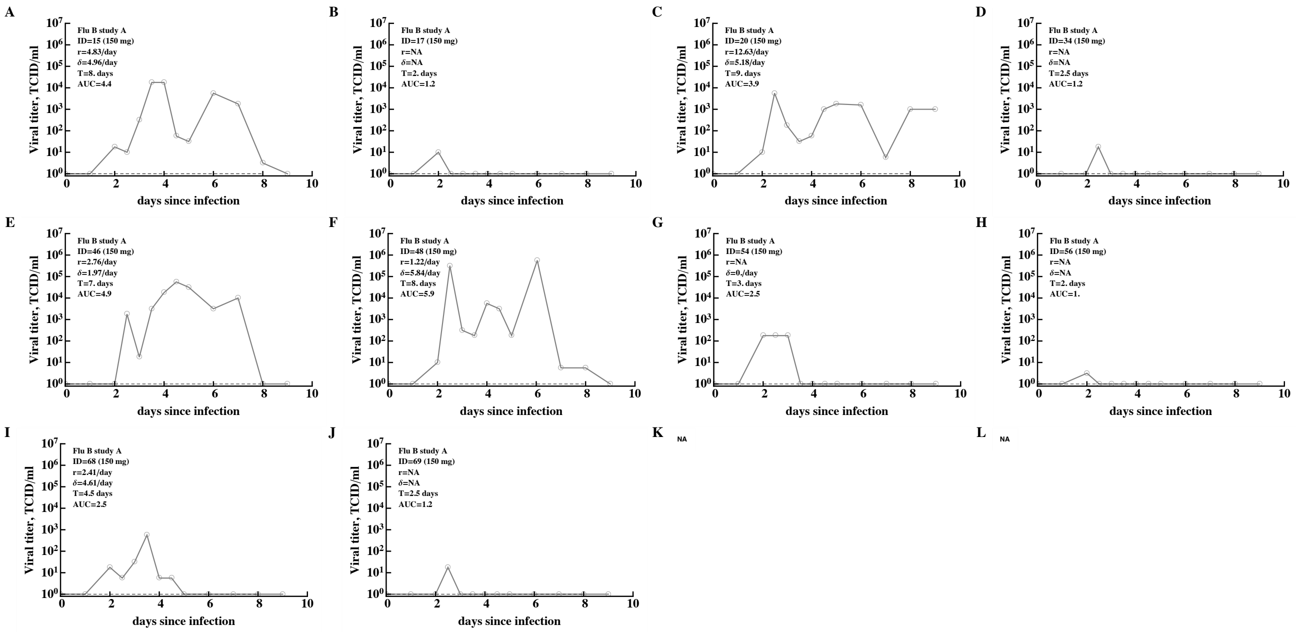

Figure S8: Viral shedding titers for individual volunteers from Flu B study A [13] treated with 150mg of oseltamivir. Volunteers excluded from the analysis as uninfected have the following IDs: 11, 26, 27, 29, 38, 40, 42, 50, 61, 63. See Figure S6 for more detail.

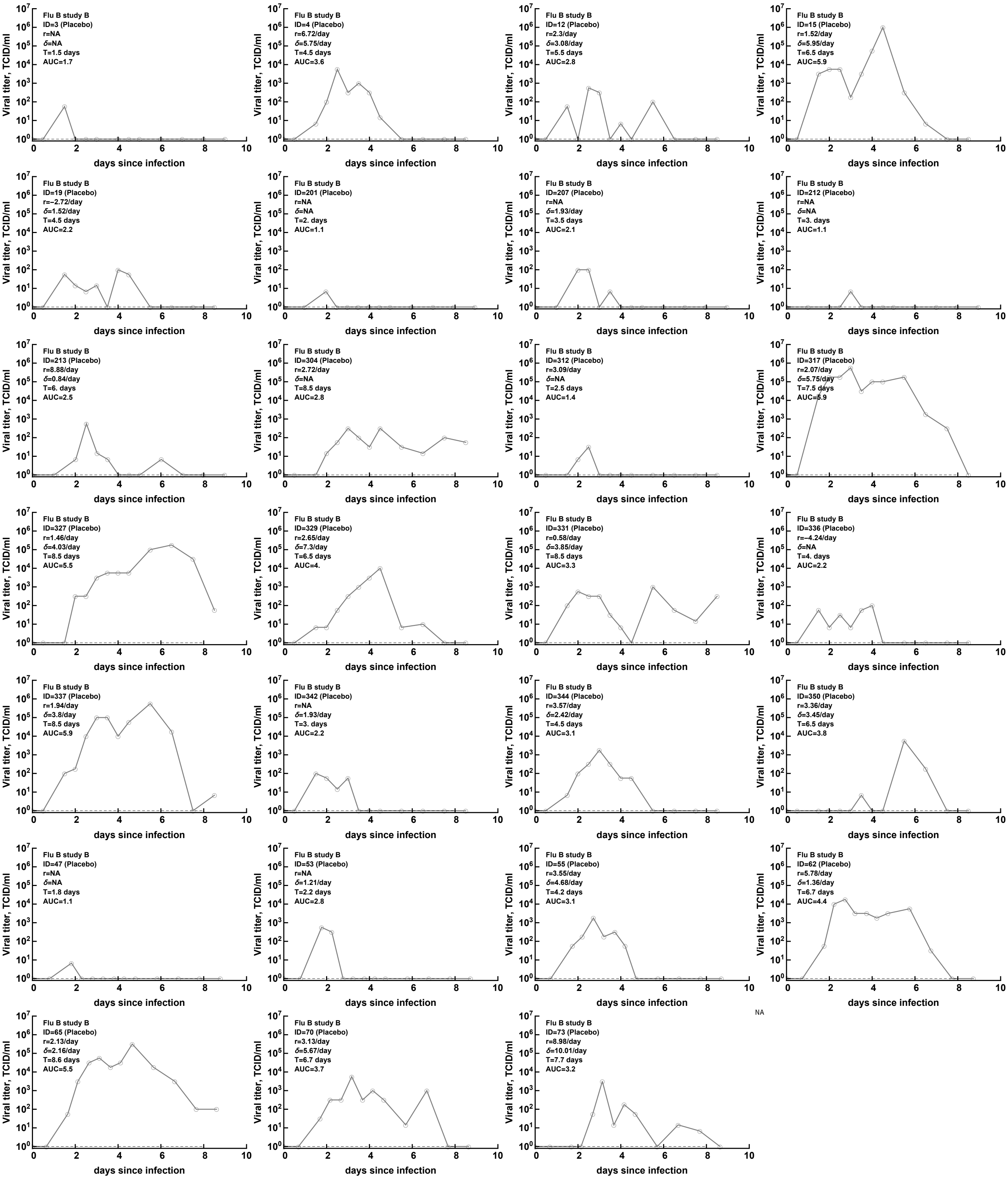

Figure S9: Viral shedding titers for individual volunteers from Flu B study B [13]. These are data for placebo-treated volunteers including the volunteer ID, duration of infection, viral growth and viral decline rates, duration of infection, and the total viral sheeting (AUC). Volunteers excluded from the analysis as uninfected have the following IDs: 8, 17, 204, 303, 308, 314, 319, 324, 346, 49, 58, 67.

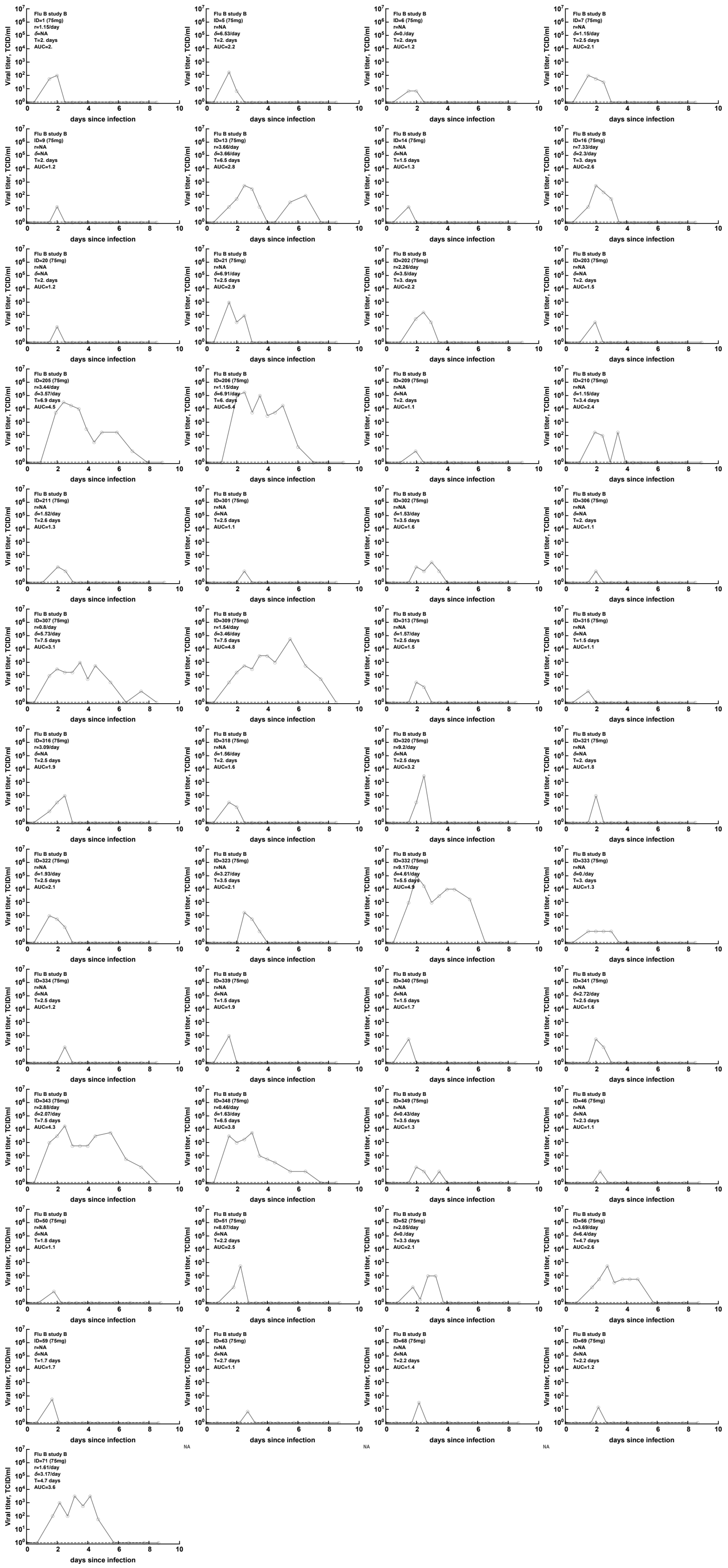

Figure S10: Viral shedding titers for individual volunteers from Flu B study A [13] treated with 75mg of oseltamivir. Volunteers excluded from the analysis as uninfected have the following IDs: 2, 10, 11, 18, 208, 214, 215, 305, 310, 311, 325, 326, 328, 330, 335, 338, 345, 347, 351, 48, 54, 57, 60, 61, 64, 66, 72, 74, 75. See Figure S9 for more detail.

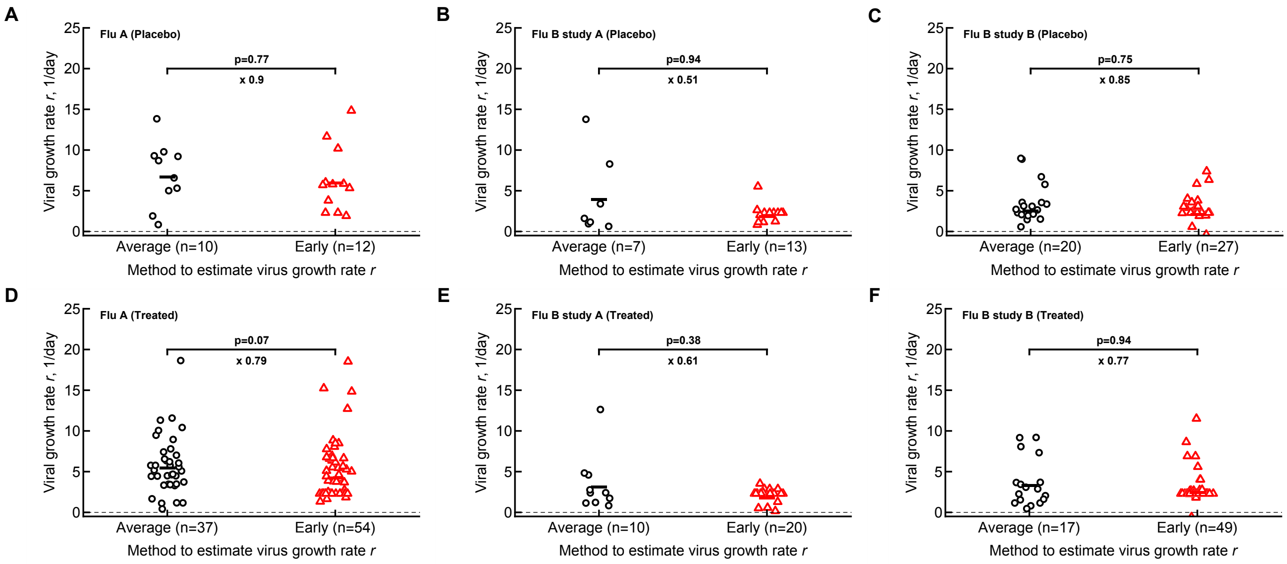

Figure S11: No difference between the average viral growth rate and the early viral growth rate in all groups of volunteers. We calculated the early virus replication rate by either only including viral shedding data prior to peak that are above the limit of detection ("Average" category) or by including all data points prior to peak including data at the limit of detection ("Early" category). Panels A&D show the Influenza A analyses. Panels B&E show the Influenza B study A analyses. Panels C&F show the Influenza B study B analyses. The small black line in each of the columns of data show the median value for that analysis. The number of volunteers, n, analyzed in each of the trial and tests is shown on the x-axis of each graph. P-values were calculated using the Mann-Whitney test.

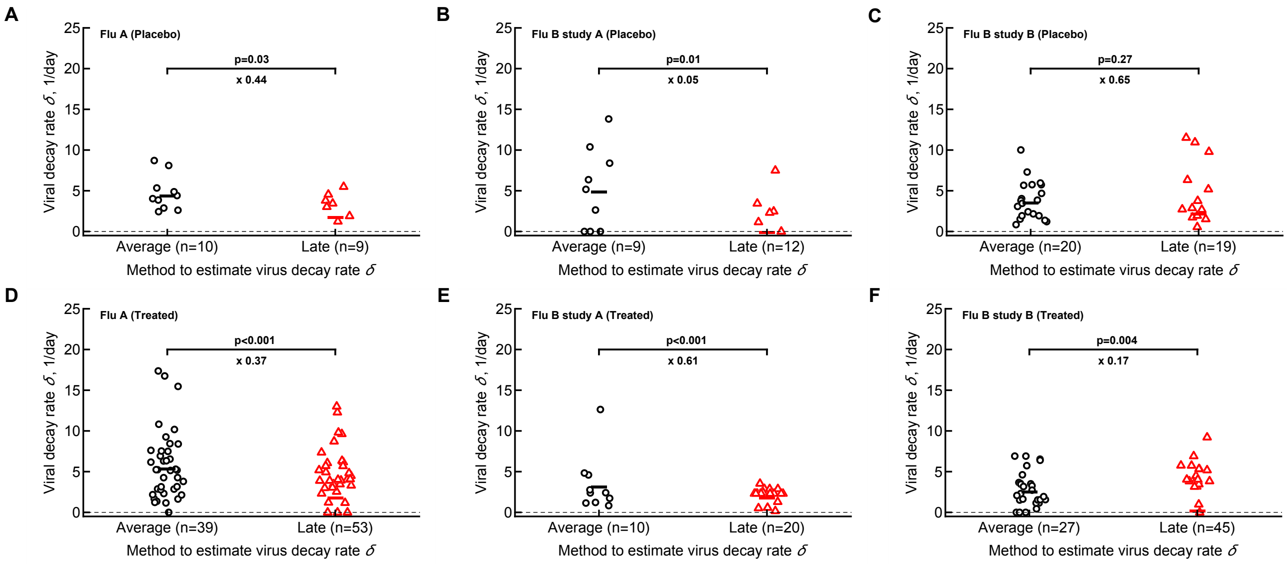

Figure S12: Most volunteer groups show a slower late viral decay rate as compared to the average decay rate. We calculated the late virus decline rate by either only including viral shedding data after the peak that are above the limit of detection ("Average" category) or by including all data points after the peak including one first data point at the limit of detection ("Late" category). Panels A&D show the Influenza A analyses . Panels B&E show the Influenza B study A analyses. Panels C&F show the Influenza B study B analyses. The small black line in each of the columns of data show the median value for that analysis. The number of volunteers, n, analyzed in each of the trial and tests is shown on the x-axis of each graph. P-values were calculated using the Mann-Whitney test.

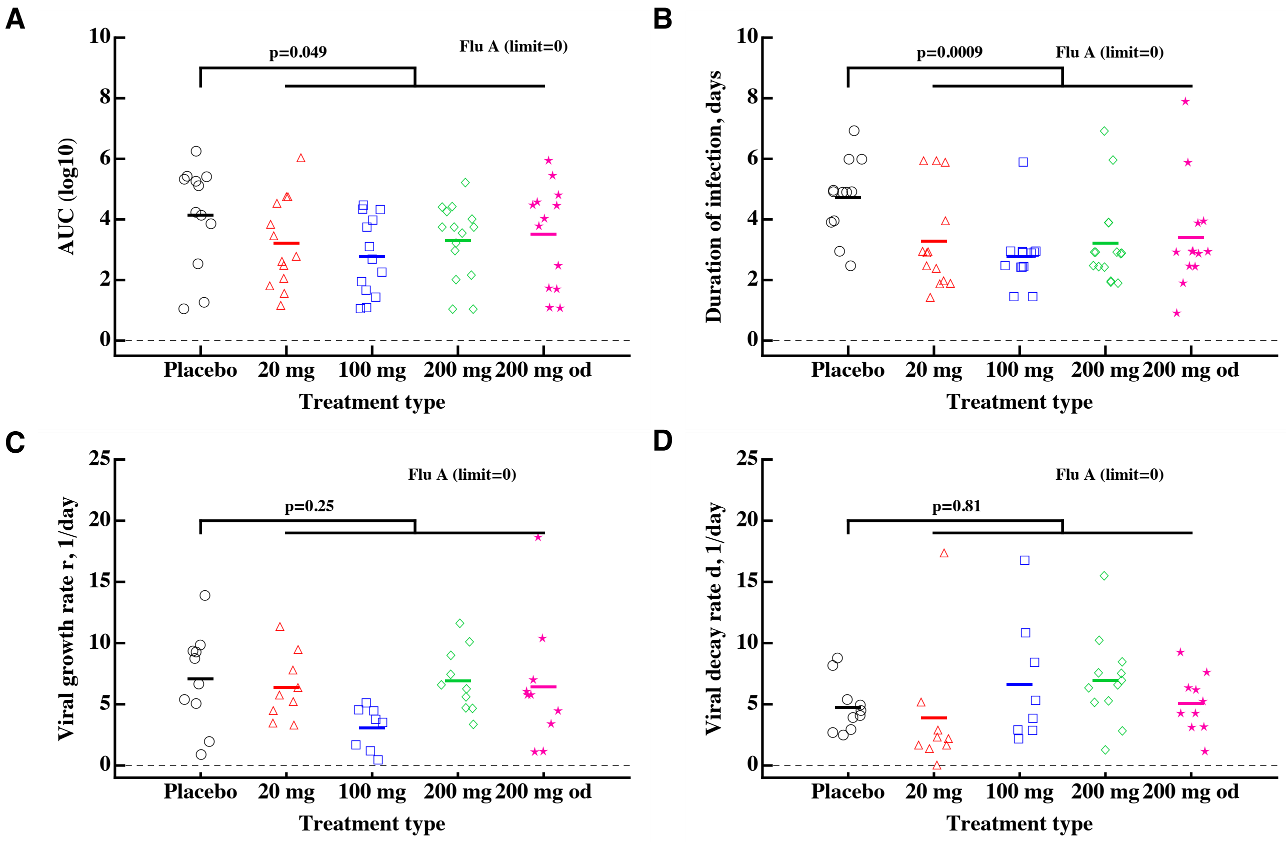

Figure S13: Impact of oseltamivir treatment type (dose) on the total viral shedding (AUC, A), duration of infection (B), viral growth (C) and decline (D) rates for individuals in Flu A clinical trial [12]. The differences in estimated parameters between treatments with different drug concentrations and/or schedules were not significant (judged by ANOVA, $p > 0.09$ for all panels). See Figures 3 and 4 for more detail.

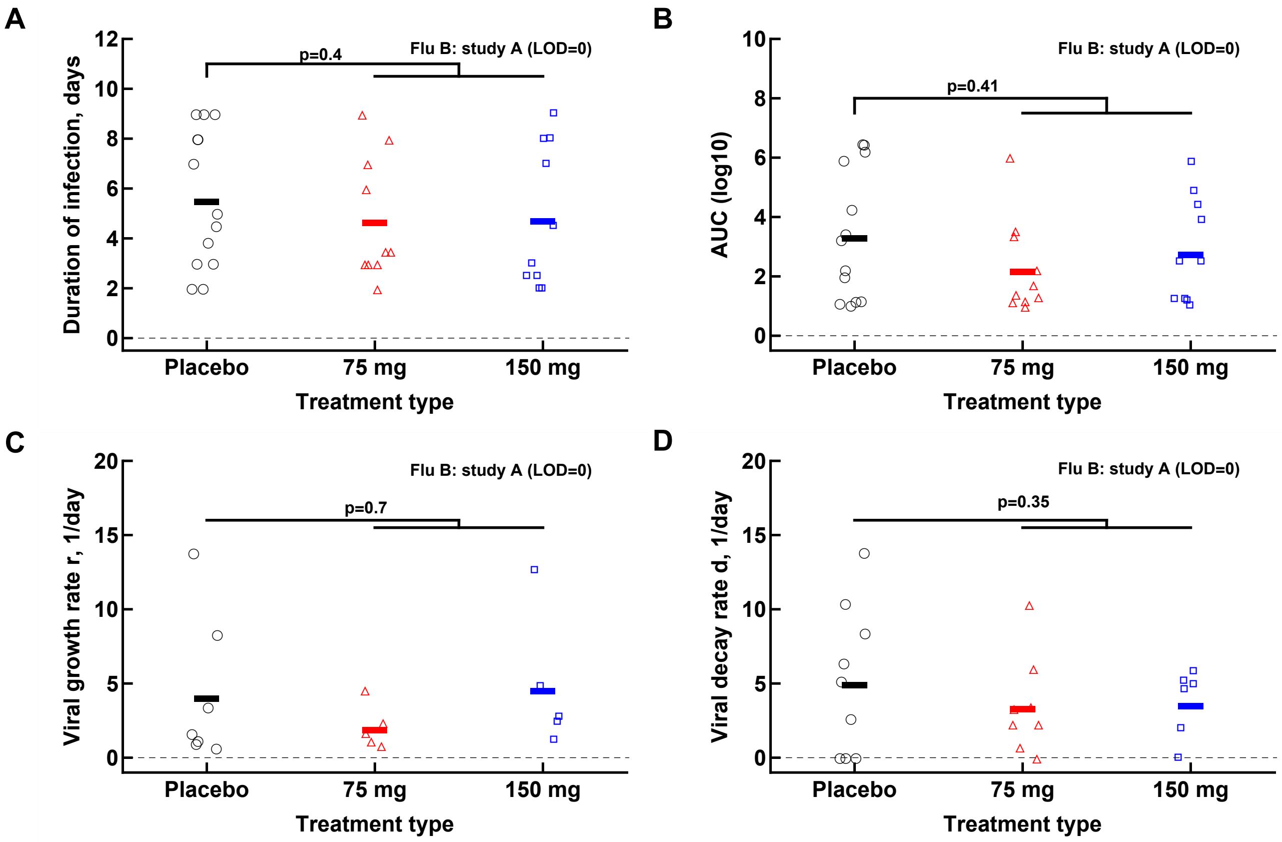

Figure S14: Impact of oseltamivir treatment type (dose) on the total viral shedding (AUC, A), duration of infection (B), viral growth (C) and decline (D) rates for individuals in Flu B study A clinical trial [13]. The differences in estimated parameters between treatments with different drug concentrations and/or schedules were not significant (judged by ANOVA, $p > 0.25$ for all panels).
